# A Highly Compliant Serpentine Shaped Polyimide Interconnect for Front-End Strain Relief in Chronic Neural Implants

**DOI:** 10.3389/fneur.2013.00124

**Published:** 2013-09-12

**Authors:** Viswanath Sankar, Justin C. Sanchez, Edward McCumiskey, Nagid Brown, Curtis R. Taylor, Gregory J. Ehlert, Henry A. Sodano, Toshikazu Nishida

**Affiliations:** ^1^Department of Electrical and Computer Engineering, University of Florida, Gainesville, FL, USA; ^2^Department of Biomedical Engineering, University of Miami, Coral Gables, FL, USA; ^3^Department of Mechanical and Aerospace Engineering, University of Florida, Gainesville, FL, USA

**Keywords:** neuroprosthetics, brain-machine interface, flexible microelectrode array, strain relief, high compliance electrode cable

## Abstract

While the signal quality of recording neural electrodes is observed to degrade over time, the degradation mechanisms are complex and less easily observable. Recording microelectrodes failures are attributed to different biological factors such as tissue encapsulation, immune response, and disruption of blood-brain barrier (BBB) and non-biological factors such as strain due to micromotion, insulation delamination, corrosion, and surface roughness on the recording site (1–4). Strain due to brain micromotion is considered to be one of the important abiotic factors contributing to the failure of the neural implants. To reduce the forces exerted by the electrode on the brain, a high compliance 2D serpentine shaped electrode cable was designed, simulated, and measured using polyimide as the substrate material. Serpentine electrode cables were fabricated using MEMS microfabrication techniques, and the prototypes were subjected to load tests to experimentally measure the compliance. The compliance of the serpentine cable was numerically modeled and quantitatively measured to be up to 10 times higher than the compliance of a straight cable of same dimensions and material.

## Introduction

Recent clinical trials have successfully demonstrated that Brain-Machine Interfaces (BMIs) can restore the lost communication and control in humans affected with a variety of neurological disorders (5–10). Though these studies have shown proof of concept of BMI function, there is still the challenge of building an ideal BMI system that is capable of obtaining high quality neural signals for chronic durations (10+ years), which is a desirable requirement for clinical deployment. The temporal degradation of signal quality in chronically implanted microelectrode neural interfaces is attributed to both biotic factors such as tissue encapsulation, immune response, and disruption of blood-brain barrier (BBB) and abiotic factors such as insulation delamination, corrosion, surface roughness on the recording site, and strain due to micromotion (1–4).

Strain due to micromotion is identified as one of the potential abiotic contributors of failure mechanisms for long-term neural implants. Histological studies (11, 12) report that the strain induced immune response caused by the rigid tethering of the electrode to the skull showed an increase in microglial activity in the implanted tissue as compared to untethered electrodes. This increased tissue response and continuous proliferation of microglial cells around the electrode can be detrimental to the well being of the neurons in the vicinity. Histological studies (13) report that upregulation of microglial biomarker ED1 was accompanied by reduction in neurons and nerve cell fibers surrounding the implant. This suggests a correlation between increased tissue response and reduced signal reliability.

When the electrode substrate is secured to the skull during implantation, it results in a rigid tethering of one end of the electrode, while the other end of the electrode, the tip, and the brain are free to move with respect to each other. The brain experiences displacements on the order of microns to millimeters driven by physiological, behavioral, and mechanical sources (14). It has been observed that the brain micromotion in anesthetized rats due to respiratory pulsation is on the order of 10–30 μm, and due to vascular pulsation is about 2–4 μm (15). This is relevant since micromotion of the brain with respect to the skull (relative micromotion) is expected to exert a force vector on the cortical tissue via the implanted electrode. The strain due to the force acting on the rigid back end is transferred along the probe shank and displaces the electrode tip within the brain tissue and may have biotic ramifications through a mechanical inflammatory process with consequences such as promoting more upregulation of microglial cells.

Numerical studies have shown that electrodes with low Young’s modulus material or redefined geometry for high compliance can provide front-end strain relief. Mechanical modeling of tethering induced strain for silicon and polymer electrodes (16) show that a rigidly tethered silicon shank transfers significant strain to the surrounding brain tissue and favor displacement of tip. Finite element analysis of a polyimide array with respect to a rigid silicon microelectrode array of same dimensions conducted (17, 18) show a front-end strain relief of 65–94%.

Polymers such as polyimide and parylene-C, with their low Young’s modulus values and good biocompatibility have been the choice of researchers for electrode substrate material. In the past decade, a number of groups have developed microelectrode arrays with polyimide as the electrode structural material (19–22), and parylene-C as the structural material (23, 24).

In terms of design modification for front-end strain relief, the first effort (25) included a single long slender gold wire as a cable between the probe and the external connection. The design limited the electrode displacement to 10 μm for 1 mm relative micromotion of the brain. Of the several electrode array configurations reported by (19), one included an “S”-shaped curve for incorporating strain relief in the cable. Recently, the serpentine shaped silicon cable showed 50% stress reduction compared to a straight silicon cable of the same dimension (26). More recently, neural probes and cables with different meandering geometries are being reported with improved compliance and better stress relief (27, 28).

In this paper, we describe the design, fabrication, and detailed mechanical characterization and numerical studies of highly compliant serpentine shaped polyimide modular electrodes. Modular electrodes are the fourth generation polyimide electrodes developed at the University of Florida after the generation 1 flexible polymer substrate electrodes (29), the generation 2 amplifier integrated microelectrode arrays (30), and the generation 3 Pyrex^®^ supported amplifier integrated microelectrode arrays. These electrodes have two modules: (i) a rigid silicon platform serving as a stage for the connector and future electronics, and (ii) a serpentine shaped flexible polyimide cable that interfaces the recording tungsten microwire array and the rigid module. The serpentine shaped cable possesses a higher compliance than a straight polyimide cable, which can provide better front-end strain relief.

## Materials and Methods

### Flexible cable and rigid module design

**Figure 1:**
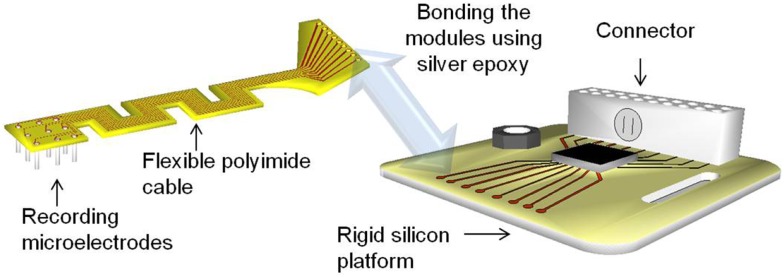
**Illustration of the modular electrode design**. Image not drawn to scale.

The modular electrodes include two modules – a rigid silicon module serving as a platform for electronics and connector, and a flexible polyimide module serving as the front-end cable between the microwires and the electronics. The modules may be fabricated independently and then bonded together using conductive silver epoxy paste, and secured and hermetically sealed with underfill epoxy. This kind of modular approach allows parallel fabrication of the modules, thereby reducing the processing time and increasing the yield. Figure 1 shows the conceptual drawing of the modular electrode design.

The flexible polyimide cable module is designed to have a serpentine structure as shown in Figure 1. The meanders in the design result in an increase in the effective length compared with a straight electrode of the same overall size. This provides higher compliance and better strain relief than its straight counterpart. At the same time, the overall form factor of the cable is still maintained the same for facilitating implantation. Furthermore, the new geometry enables placing the recording microwires in a two dimensional transverse fashion, with nine electrodes being placed in a 3 × 3 array.

### Flexible cable and rigid module fabrication

**Figure 2:**
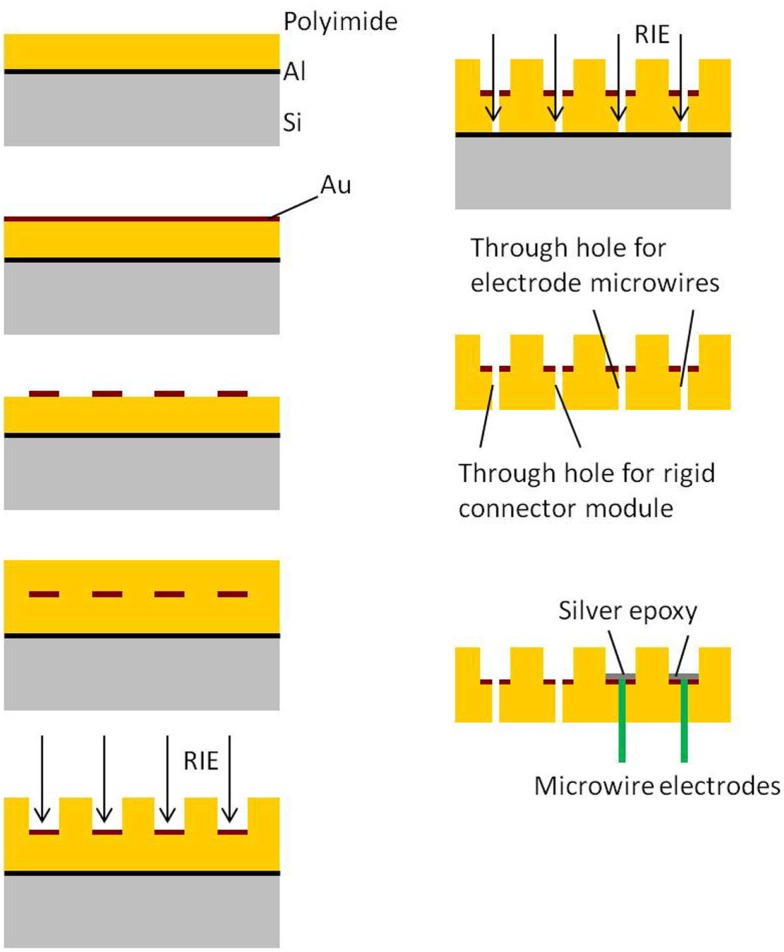
**Process flow for 2D transverse flexible cable module**.

Processing and packaging steps involved in the fabrication of the 2D cables are shown in Figure 2. All the steps involved in the processing are done on a rigid 4″ silicon wafer. The process begins with the spin deposition of 20 μm thick layer of polyimide on top of a sacrificial aluminum film. Next, a thin film (∼2000 Å) of gold is sputter deposited and lithographically patterned to define the conductive traces. The top insulation is provided by spinning a layer of 20 μm thick polyimide over patterned gold. The top polyimide is plasma etched using a reactive ion etcher (RIE) to expose gold bondpads surrounding the via holes. Also the through holes are obtained by etching off bottom polyimide underneath them. Finally the device is released from the Si wafer by etching the sacrificial aluminum layer through anodic dissolution process.

**Figure 3:**
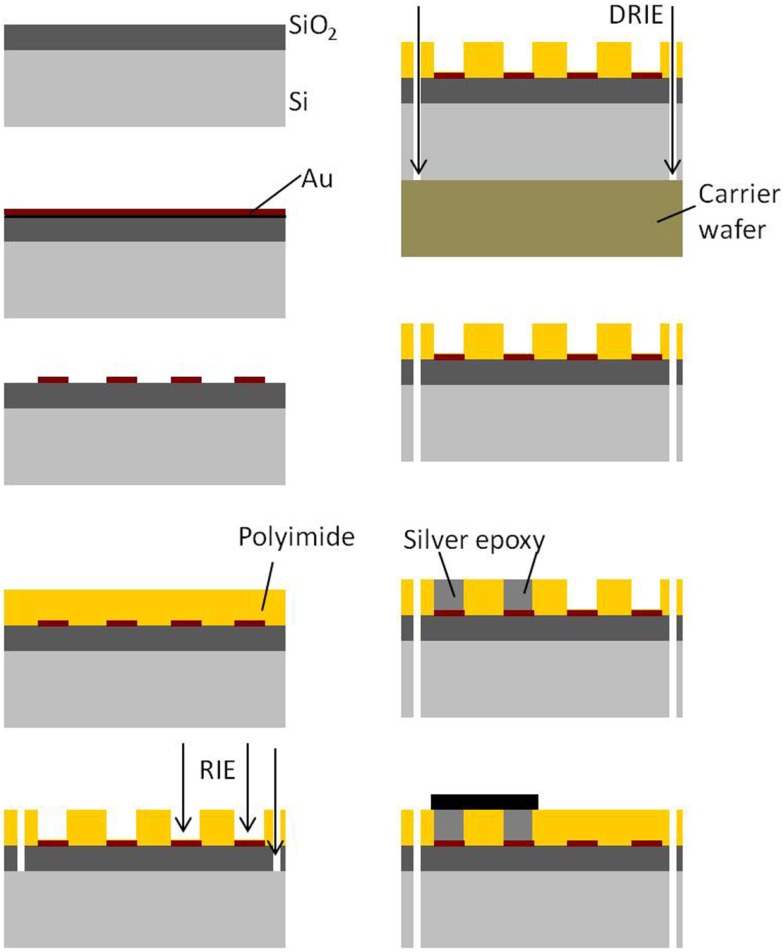
**Process flow for rigid electronics and connector module**.

The processing and packaging steps involved in the fabrication of the rigid electronics and connector module are shown in Figure 3. All the steps are carried out on a 4″ silicon wafer. First, a thin film of SiO_2_ is deposited on silicon. Similar to the process flow of the flexible module, a thin film (∼2000 Å) of gold is sputter deposited on the oxide layer and lithographically patterned to define the conductive traces. The top insulation is deposited by spinning a layer of 20 μm thick polyimide over the patterned gold. The top polyimide is plasma etched using RIE to expose gold bondpads for the connector and the electronics. The hole for the ground screw and the slot hole are obtained by etching off the polyimide and oxide underneath them. Finally, the silicon underneath the screw hole, slot hole, and the surrounding device outline is etched off using deep reactive ion etch (DRIE) to separate the module. Once the modules are separated into single pieces, the connector and the nut are attached using conductive silver epoxy.

**Figure 4:**
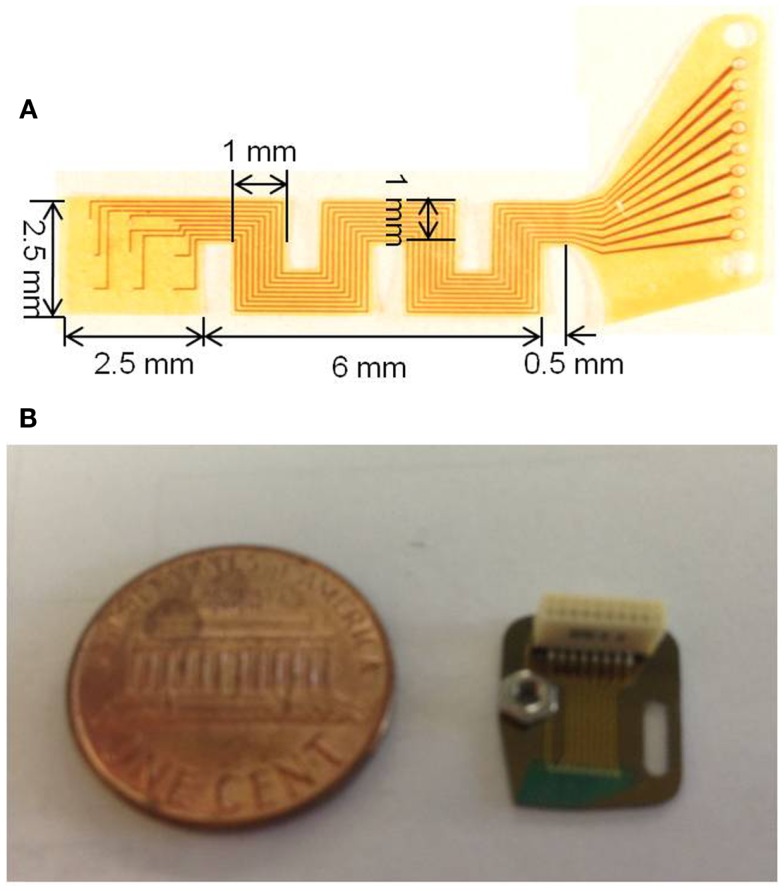
**(A)** Photograph of the micro-fabricated serpentine polyimide cable prior to the attachment of the tungsten microwires and **(B)** photograph showing the fabricated silicon rigid module packaged with the Omnetics connector and the ground screw compared against a one cent coin.

Photographs of the fabricated serpentine cable prior to attachment of the microwires and rigid module are shown in Figures 4A,B, respectively.

### Analytical and numerical modeling of cable compliance

#### Analytical analysis

**Figure 5:**
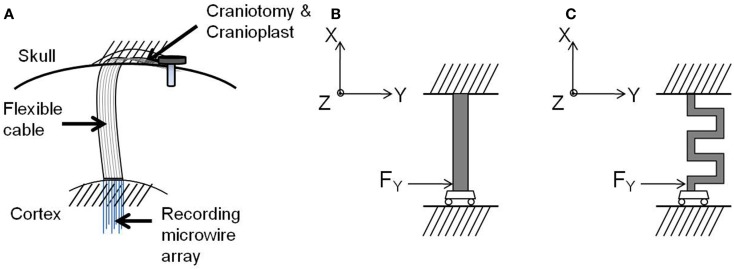
**(A)** Schematic drawing of the electrode implanted into the brain, **(B)** free-body diagram of the straight cable, and **(C)** free-body diagram of the serpentine cable. Images not drawn to scale.

After implantation, one end of the electrode cable is attached firmly to the skull resulting in a rigid tethering, while the other end containing the recording microelectrode array is inserted into the cortex, as shown in Figure 5A. The natural respiration and vascular pulsation along with head movement of the animal may result in the micromotion of the brain with respect to the skull. The brain exhibits motion in all the three axes. The translational movement of brain with respect to the skull in *x* and *y* axes exerts a force both radial and tangential to the electrode cable (17). In addition, the translational movement of the brain in the axis orthogonal to the plane of the electrode (*z*-axis) contributes to the shearing of the cable. However, for this analysis, three assumptions were made:

1. the rotational motion of the brain and hence the torsion of the electrode is not considered,
2. the force acting on the electrode is assumed to be a point load concentrated at the cortex end of the electrode cable, and
3. the cable is fixed in the vertical direction at the surface of the skull.

Guided by these boundary conditions and assumptions, a beam model was employed for the electrode cable. According to the beam model, the cable is considered as a clamped-guided beam, which is fixed at the skull and free to move in *x*, *y*, and *z* axes along the cortex, and a concentrated force is acting at the guided end. Figures 5B,C show the free-body diagrams of the straight cable and serpentine cable respectively.

Closed-form theoretical expressions for classic serpentine springs (31) were used for calculating the spring constants and compliances of the serpentine cable in *x*, *y*, and *z* directions.

The stiffness equations in the *x*, *y*, and *z* axes for the serpentine cable are given by (31),

(1) $$K_{x}^{\text{C}}={\left[\frac{\left(N+1\right)l_{\text{o}}^{3}}{6EI_{z_{\text{o}}}}+\frac{\left(N+1\right)l_{\text{o}}^{2}l_{\text{p}}}{2EI_{z_{\text{p}}}}\right]}^{-1},$$

where *N* is the number of meanders, *l_o_* is the length of the meander element perpendicular to the *x* and *z* axes (*m*), *l_p_* is the length of the meander element parallel to the *x*-axis, $I_{z_{o}}$ is the moment of inertia with reference to the *z*-axis of the meander element section perpendicular to *x* and *z* axes (*m*^4^), and $I_{z_{p}}$ is the moment of inertia with reference to the *z*-axis of the meander element section parallel to *x*-axis (*m*^4^).

(2) $$K_{\delta_{y}}^{\text{C}}=\frac{K_{y\theta_{z}}^{\text{C}}K_{\theta_{z}y}^{\text{C}}K_{y}^{\text{C}}}{K_{y}\theta_{z}^{\text{C}}K_{\theta_{z}y}^{\text{C}}-K_{y}^{\text{C}}K_{\theta_{z}}^{\text{C}}},$$

where,

(3) $$\begin{array}{ll} K_{y}^{\text{C}} & ={\left[\frac{{\left(2\left(N+2\right)l_{\text{p}}\right)}^{3}}{3EI_{z_{\text{p}}}}+\frac{\left(8N^{3}+36N^{2}+55N+27\right)l_{\text{p}}^{2}l_{\text{o}}}{3EI_{z_{\text{o}}}}\right]}^{-1}\;\;, \end{array}$$

(4) $$\begin{array}{ll} K_{y\theta_{z}}^{\text{C}} & =K_{\theta \mathrm{zy}}^{\text{C}}={\left[\frac{2\left(N^{2}+3N+4\right)l_{\text{p}}l_{\text{o}}}{EI_{z_{\text{o}}}}+\frac{{\left(2N+2\right)}^{2}l_{\text{p}}^{2}}{EI_{z_{\text{p}}}}\right]}^{-1}, \end{array}$$

(5) $$\begin{array}{ll} K_{\theta_{z}}^{\text{C}} & ={\left[\frac{2\left(N+2\right)l_{\text{p}}}{EI_{z_{\text{p}}}}+\frac{2\left(N+1\right)l_{\text{o}}}{EI_{z_{\text{o}}}}\right]}^{-1}, \end{array}$$

and,

(6) $$K_{\delta_{z}}^{\text{C}}=\frac{K_{z\theta_{y}}^{\text{C}}K_{\theta_{y}z}^{\text{C}}K_{z}^{\text{C}}}{K_{z\theta_{y}}^{\text{C}}K_{\theta_{y}}z^{\text{C}}-K_{z}^{\text{C}}K_{\theta_{y}}^{\text{C}}},$$

where,

(7) $$\begin{array}{llll} K_{z}^{\text{C}} & =\left[\frac{{\left(2\left(N+2\right)l_{\text{p}}\right)}^{3}}{3EI_{y_{\text{p}}}}+\frac{\left(N+1\right){\left(l_{\text{o}}\right)}^{3}}{6EI_{y_{\text{o}}}}+\frac{\left(N+1\right){\left(l_{\text{o}}\right)}^{2}l_{\text{p}}}{GJ_{\text{p}}}\right.\; & & \;\\ & {\left.\;+\frac{\left(8N^{3}+36N^{2}+55N+\text{27}\right)l_{\text{p}}^{2}l_{\text{o}}}{3GJ_{\text{o}}}\right]}^{-1}, \end{array}$$

(8) $$\begin{array}{ll} K_{\theta_{y}z}^{\text{C}} & =K_{z\theta y}^{\text{C}}{\left[\frac{2\left(N^{2}+3N+4\right)l_{\text{p}}l_{\text{o}}}{GJ_{\text{o}}}+\frac{2{\left(N+2\right)}^{2}l_{\text{p}}^{2}}{EI_{y_{\text{p}}}}\right]}^{-1}, \end{array}$$

(9) $$\begin{array}{ll} K_{\theta_{y}}^{\text{C}} & ={\left[\frac{2\left(N+2\right)l_{\text{p}}}{EI_{y_{\text{p}}}}+\frac{2\left(N+1\right)l_{\text{o}}}{GJ_{\text{o}}}\right]}^{-1}, \end{array}$$

where $I_{y_{o}}$ is the moment of inertia with reference to the *y*-axis of the meander element section perpendicular to *x* and *z* axes (*m*^4^), and $I_{y_{p}}$ is the moment of inertia with reference to the *y*-axis of the meander element section parallel to *x*-axis (*m*^4^), *J_o_* is the cross-sectional torsion factor of the meander element perpendicular to *x* and *z* axes (*m*^4^), *J*_p_ is the cross-sectional torsion factor of the meander element parallel to *x*-axis (*m*^4^), and *G* is the shear modulus (Pa).

The spring constants for the straight cables were calculated using the standard stiffness equations for a clamped-guided beam (32). The stiffness equations in *x*, *y*, and *z* axes are given by,

(10) $$\begin{array}{ll} K_{x} & =\frac{\mathrm{Ehw}}{L}, \end{array}$$

(11) $$\begin{array}{ll} K_{y} & =\frac{\mathrm{Eh}w^{3}}{L^{3}}, \end{array}$$

and

(12) $$K_{z}=\frac{Eh^{3}w}{L^{3}}$$

Since the stress due to complex mechanical motions and biological and chemical reactions are not considered for this analysis, the standard stiffness equations may be used to estimate the straight cable compliance. However, it should be noted that in a real time *in vivo* condition, these equations may not completely model the cable compliance.

Table 1 gives the dimensions of the straight and the serpentine cables used in the analytical and numerical estimation of compliance.

**Table 1:** **Dimensions of the straight and serpentine cables used in the analytical and numerical analysis**.

Dimension	Straight cable	Serpentine cable
Length	6 mm	6 mm
Width	2.7 mm	2.5 mm
Height	40 μm	40 μm
Number of meanders	–	2
Length of meander	–	1 mm
Width of meander	–	1 mm
Length of end spans	–	0.5 mm

##### Material uncertainty

In the literature, the reported Young’s modulus of polyimide ranges from 2.793 to 15 GPa depending on the formulation. Table 2 gives a list of all Young’s modulus values for polyimide obtained from the literature.

**Table 2:** **List of Young’s modulus values of polyimide obtained from literature**.

Reference	Young’s modulus of polyimide (GPa)
Rousche et al. (19)	2.793
Dupont Kapton B technical datasheet^a^	3.0
Dolbow and Gosz (33)	7.5
HD microsystems PI2611 process guide^b^	8.5
Dolbow and Gosz (33)	8–15

^a^http://www2.dupont.com/Kapton/enUS/assets/downloads/pdf/KaptonB.pdf

^b^http://hdmicrosystems.com/HDMicroSystems/enUS/pdf/PI-2600ProcessGuide.pdf

The compliance was calculated for all of the values listed in Table 2 for both the straight and the serpentine cables. Mean and standard deviations were obtained for the calculated compliance values to account for the error due to variation in material property.

#### Numerical analysis

Following the analytical analysis, a numerical analysis was performed. A finite element model was developed for the compliances of straight and serpentine beams using ABAQUS simulation tool. The 2D Shell element was used for the analysis. The mesh shape is quadrilateral element for straight cables and quadrilateral and triangular elements for serpentine cables. The mesh size is within the ABAQUS default size range (min: 1.3e−6 and max: 0.0013), and the mesh number is 50 elements for straight cables and 833 elements for serpentine cables. The test for convergence employs the ABAQUS default convergence criteria values for non-linear problems as described in ABAQUS Analysis User’s Manual^1^. The boundary conditions used were encastré (no rotational or translational motion in any axis) for fixed end and no rotational motion, only translational displacement in all axes for the guided end. Simulations were performed for all of the Young’s modulus values of polyimide given in Table 2 and the results were averaged and the error was calculated.

### Experimental measurement cable compliance

#### Experimental measurement of the in-plane (*x*-axis) cable compliance

The in-plane (or *x*-axis) stiffness of the micro-fabricated straight and serpentine cable electrodes was experimentally measured using an Instron^®^ 5900 series mechanical testing system. The cables were subjected to in-plane tensile stress, and the extension in the axial direction was measured to evaluate the stiffness in the *x*-axis. The naming of the axes can be found in Figure 5.

The cables were suspended between two vertically positioned clips that were connected to load cells, which run across two load frames. The top frame is movable while the bottom remains fixed. Extension control testing method was used to measure the compliance of the cables. In this testing method, the cable is subjected to a known extension (from *x*_0_ to *x*_1_ in steps of Δ*x*) for a given period of time. From Hooke’s law, *F* = *kx*, a change in displacement is induced by a change in the force. The corresponding change in the force Δ*F* is measured. The measured change in force is plotted against the change in displacement, and the slope of the resulting curve is calculated to find the stiffness of the cable.

**Figure 6:**
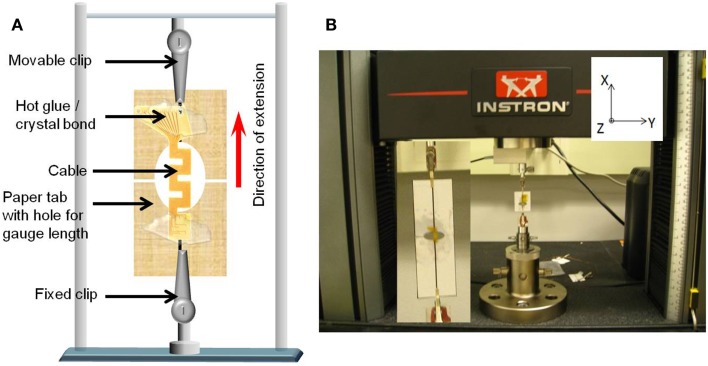
**(A)** Schematic diagram of the paper mounted cable suspended between the two vertical clips connected to the load cells and **(B)** photograph of the cable mounted on the paper tab connected to the clips of the Instron^®^ mechanical testing system. Inset shows the closer view of the mounted cable.

In order to ensure the consistency of cable length with the analytical and numerical calculations, the cables were mounted on paper tabs with holes in the center using hot glue or crystal bond. The size of the holes corresponded to the length of the cable. The paper tabs were then attached to the two vertical clips. Once attached, the paper was cut at the center to prevent any additional loading due to the paper. This set up has more control on the gage length of the cable. Figure 6A shows the schematic drawing of the paper mounted cable suspended between the two vertical clips, and Figure 6B shows a photograph of the cable mounted on the paper tab connected to the clips of the Instron^®^ mechanical testing system.

The straight cables were extended from 0 to 50 μm. The maximum load limit set on the straight cables was 300 mN. The serpentine cables were extended from 0 to 40 μm, and the maximum load limit on them was 100 mN. One sample of straight cable and one sample of serpentine cable were measured for stiffness and 10 trials were performed on each sample for consistency. Mechanical deformation such as necking was not observed in the in-plane stiffness measurement experiments as the load was applied within the elastic region. Also no buckling was observed since no compressive stress was applied.

#### Experimental measurement of the out-of-plane (*y*-axis) cable compliance

The out-of-plane (or *y*-axis) stiffness of the micro-fabricated straight and serpentine cable electrodes was experimentally measured using a Hysitron^®^ TriboIndenter (TI 900). The instrument has a lab noise floor displacement resolution of 1 nm, and force resolution of 100 nN. The cables were loaded by lowering the indenter tip down for a preset load limit and a depth limit, and the change in the displacement was measured as a function of the change in the load to evaluate the stiffness. A diamond (fluid cell) conical tip of nominal radius 20.1 μm was used for loading the samples. The loading conditions included a preload force of 0.3 μN with a triangular ramp of 100 nm/s and a maximum displacement of 4.8 μm.

##### Sample preparation

In order to facilitate vertical loading of the cables along its thin *y*-axis, the cables were mounted vertically on a glass slide and secured with two magnet pieces and steel nuts on either side. This set up ensured vertical standing of the cable without much movement. It was also confirmed that the nuts and magnets remain steady during the loading of the tip. One sample of straight cable, and one sample of serpentine cable were prepared for the experiment.

### Experiment setup

**Figure 7:**
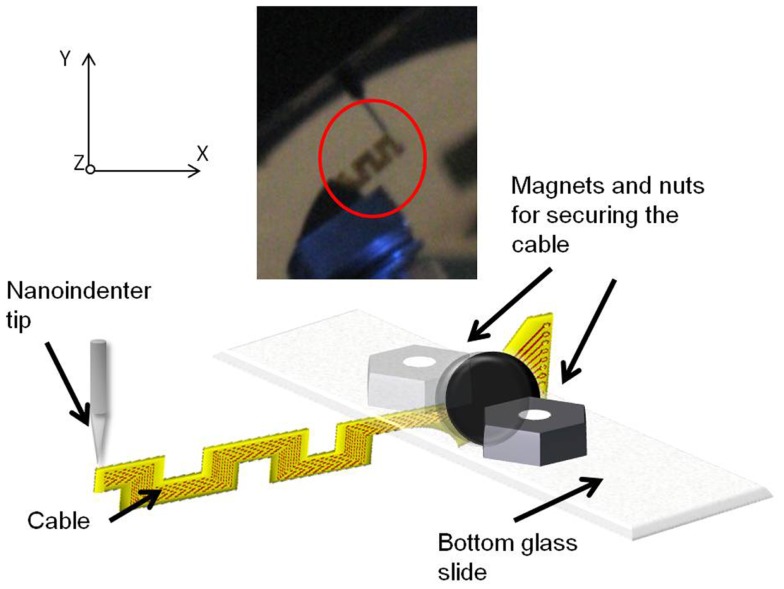
**Schematic diagram showing the vertical loading of the serpentine cable in *y*-axis by the nanoindenter tip**. Inset showing the photograph of the cable and the nanoindenter tip.

The mounted sample was introduced into the TriboIndenter chamber, and placed directly under the conical tip. The system was allowed to thermally stabilize for 10 min. First, air indent calibration was done. Next, the initial position (or the zero point) was calibrated by slowly lowering the tip and establishing a contact with the cable at a force<2 μN. After calibration, the cable was loaded by lowering the tip further down for a preset load and depth limit. The maximum load limit was set at 25 μN, and the maximum depth was set at 4700 nm. The change in the displacement was measured as a function of the change in the load, and the slope of the resulting plot was calculated to obtain the compliance value. Five trials were performed on each sample for consistency and statistics. Figure 7 shows the schematic drawing of the vertical loading of the serpentine cable in *y*-axis by the conical nanoindenter tip along with a photograph. The *y*-axis stiffness measurement experiments were conducted within the elastic region of the cables and hence no tearing due to fracture was observed. Further, since the gold traces are very thin (200 nm) when compared to the polyimide film (∼40 μm thick), their contribution to the overall bending stiffness will be<1%, and hence can be neglected. The indent test was conducted at room temperature and an *in situ* optical microscope was used to align the tip with the location of indent on the sample.

#### Experimental measurement of the out-of-plane (*z*-axis) cable compliance

The out-of-plane (or *z*-axis) stiffness of the micro-fabricated straight and serpentine cable electrodes was experimentally measured using a Hysitron^®^ TriboIndenter (TI 900). The cables were loaded by lowering the indenter tip down for a preset load limit and a depth limit, and the change in the displacement was measured as a function of the change in the load to evaluate the stiffness. To minimize experimental errors, the same conical tip of nominal radius 20.1 μm used in *y*-axis measurement was used for loading the samples. Similar to *y*-axis experiments, the loading conditions included a preload force of 0.3 μN with a triangular ramp of 100 nm/s and a maximum displacement of 4.8 μm.

##### Sample preparation

Prior to the experiment, the electrode samples were prepared to facilitate the loading of the nanoindenter tip on the edge of the cable end without any damage to the electrode and the tip. The electrode substrates were mounted on a glass slide and firmly secured with hot glue at the back end. The cable end of the substrate was extended beyond the edge of the glass slide to allow free movement upon loading. A thin microscope slide was added on top of the back end to ensure flatness of the substrate. The mounted samples were then placed on the bottom plate of the nanoindenter. Since the cables are loaded vertically by the nanoindenter tip, it is required to have enough clearance in the plane of loading. Two small pieces of magnets were placed underneath the glass slide to increase the clearance of the sample in the *z*-axis.

One sample of straight cable and one sample of serpentine cable were prepared for the experiment. After completing the experiment, the samples were removed from the glass slides by heating the glue and separating the slides.

##### Experimental setup

Sample mounted on the bottom plate was introduced into the chamber of the Hysitron^®^ TriboIndenter, and placed directly under the conical tip. At first, air indent calibration was done similar to *y*-axis loading. Later, the zero point was calibrated by slowly lowering the tip and establishing a contact with the cable at a force<2 μN. After calibration, the cable was loaded by lowering the tip further down for a preset load limit and a depth limit. The maximum load limit was set at 25 μN and the maximum depth was set at 4700 nm. The change in the displacement was measured as a function of the change in the load, and the slope of the resulting plot was calculated to obtain the compliance value.

**Figure 8:**
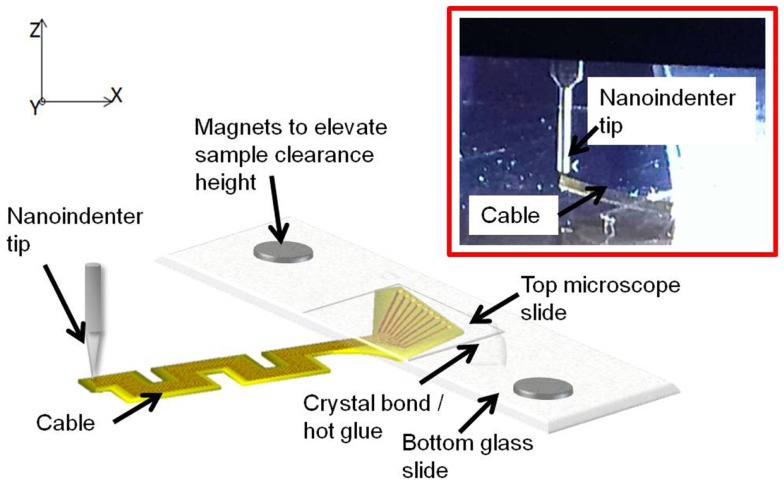
**Schematic diagram showing the vertical loading of the cable in the *z*-axis by the nanoindenter tip**. Inset shows the zoomed in photograph of the tip and the cable.

The experiment was conducted at three different points on the cable – the left most tip, the right most tip, and the center point. Five trials were performed on each measuring point for consistency and statistics. The data obtained for the right and left tips showed high non-linearity for serpentine cable. Hence only the data obtained at the center point, which were linear, were used for analysis. Figure 8 shows the schematic drawing of the vertical loading of the cable in the *z*-axis by the nanoindenter tip and a photograph of the nanoindenter tip and the cable. The *z*-axis stiffness measurement experiments were conducted within the elastic region of the cables and hence no tearing due to fracture was observed. Further, since the gold traces are very thin (200 nm) when compared to the polyimide film (∼40 μm thick), their contribution to the overall bending stiffness will be<1%, and hence can be neglected. The indent test was conducted at room temperature and an *in situ* optical microscope was used to align tip with location of indent on the sample.

#### Uncertainty analysis of the experimental error

The instrument error was included for in-plane experiments by calculating the variability in the load measurement for each measured reading based on the accuracy values (±5%) obtained from the instrument manual ^2^. The instrument error was included for out-of-plane experiments by calculating the drift in transducer displacement for the loading duration. The value used for transducer displacement drift was obtained from the instrument manual as 0.05 nm/s ^3^.

The measured values from different trials for each sample were analyzed for experimental uncertainty and the confidence interval was constructed. Two tailed *t*-test was used for confidence interval calculations since the number of trials (samples) is<30.

The confidence interval for a *t*-distribution is given by,

(13) $$\text{C.I.}\;=\bar{x}\pm t_{\alpha }∕2,n-1\frac{s}{\sqrt{n}},$$

where, $\bar{x}=\text{mean,}$ α = significance level (0.05 for 95% C.I.), *n* − 1 = number of degrees of freedom, *n* = sample size, and *s* = sample standard deviation, which is given by,

(14) $$s\;=\sqrt{\frac{s}{n-1}}\sum_{i}=1^{n}{\left(x_{i}-\bar{x}\right)}^{2},$$

## Results

Table 3 gives a summary of straight cable compliance estimated using different methods for all three axes, and Table 4 gives a summary of serpentine cable compliance estimated using different methods for all three axes. It can be observed (from the experimental results) that the compliance value of the serpentine shaped cables is at least one order of magnitude higher than the compliance of the straight cables of the same dimensions. The higher compliance or flexibility of the new serpentine shaped cables is expected to lessen the front-end strain of the electrode on the tissue. Mitigated front-end strain is expected to reduce the tissue immune response and improve the reliability of the implant’s signal recording quality.

**Table 3:** **Matrix comparing the straight cable compliance estimated through analytical, numerical, and experimental analysis**.

Type of analysis	*x*-Axis compliance (m/N)	*y*-Axis compliance (m/N)	*z*-Axis compliance (m/N)
Analytical	1.12 ± 0.67 × 10^−5^	5.54 ± 3.29 × 10^−5^	0.252 ± 0.15
Numerical	1.12 ± 0.67 × 10^−5^	2.57 ± 1.52 × 10^−4^	0.25 ± 0.149
Experimental	5.69 ± 0.48 × 10^−5^	7.43 ± 1.07 × 10^−3^	0.188 ± 0.06

**Table 4:** **Matrix comparing the serpentine cable compliance estimated through analytical, numerical, and experimental analysis**.

Type of analysis	*x*-Axis compliance (m/N)	*y*-Axis compliance (m/N)	*z*-Axis compliance (m/N)
Analytical	1.89 ± 1.12 × 10^−3^	2.7 ± 1.6 × 10^−2^	26.6 ± 15.78
Numerical	1.92 ± 1.14 × 10^−3^	9.8 ± 5.8 × 10^−3^	1.17 ± 0.69
Experimental	5.34 ± 0.19 × 10^−4^	6.8 ± 1.8 × 10^−2^	1.54 ± 0.56

**Figure 9:**
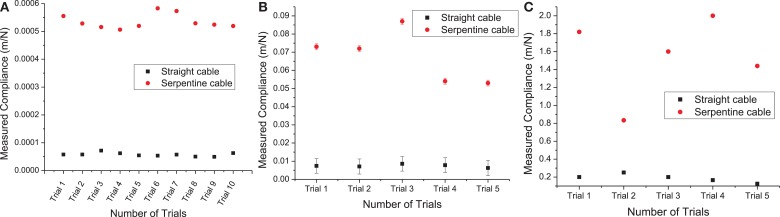
**(A)** Measured *x*-axis compliance values from straight and serpentine cables. Load measurement variability was nearly negligible (two orders of magnitude less) for straight and serpentine cables, **(B)** measured *y*-axis compliance values from straight and serpentine cables. The error bars represent the variability due to transducer displacement drift during the loading period, and **(C)** measured *z*-axis compliance values from straight and serpentine cables. Transducer displacement drift during the loading period was nearly negligible (two orders of magnitude less) for straight and (four orders of magnitude less) for serpentine cables.

The measured compliance values for the straight and the serpentine cables for *x*, *y*, and *z* axes are given in Figure 9. It can be noted from Figure 9A that the in-plane compliance values for the serpentine cable is nearly 10 times higher than that for the straight cable. Similarly, it can be observed from Figure 9B that the *y*-axis compliance of the serpentine cable is nearly one order of magnitude higher than that of straight cable. Also from Figure 9C, it is apparent that the serpentine cable has a compliance that is at least six times higher than that of the straight cable in the *z*-axis.

In addition to these experiments, the compliance of the straight and serpentine cables of reduced lengths (<6 mm) and increased widths (≥2.5 mm) were calculated using analytical methods to study the effect of variability in dimension on cable compliance. The lengths used for the analysis were 4.2, 3.84, 3.8, and 3.44 mm, and the widths used were 2.5, 3, and 3.5 mm. The Young’s modulus value of polyimide used for the analysis was 2.8 GPa. All assumptions were same as described earlier. The analytical results showed that the compliance of the serpentine cables was much higher than the straight cables in all three axes, with highest increase in the *y*-axis. Further, the compliance increased with the decrease in the cable length and increase in the cable width. The analytically calculated compliance values for straight and serpentine cables of reduced length and increased width are given in Table 5.

**Table 5:** **Matrix comparing the analytically calculated compliances for short and wide straight and serpentine cables (overall cable length<6 mm and overall cable width ≥2.5 mm)**.

Overall cable width (mm)	Overall cable length (mm)	*x*-Axis			*y*-Axis			*z*-Axis		
		Straight cable compliance (m/N)	Serpentine cable compliance (m/N)	Increase in compliance of serpentine cable (times)	Straight cable compliance (m/N)	Serpentine cable compliance (m/N)	Increase in compliance of serpentine cable (times)	Straight cable compliance (m/N)	Serpentine cable compliance (m/N)	Increase in compliance of serpentine cable (times)
2.5	4.2	1.50 × 10^−5^	6.29 × 10^−3^	419	4.24 × 10^−5^	0.034	801	0.166	24.57	148
	3.84	1.37 × 10^−5^	8.59 × 10^−3^	627	3.24 × 10^−5^	0.032	987	0.127	20.79	163
	3.8	1.36 × 10^−5^	6.04 × 10^−3^	444	3.14 × 10^−5^	0.027	860	0.122	20.92	171
	3.44	1.23 × 10^−5^	8.24 × 10^−3^	670	2.33 × 10^−5^	0.025	1072	0.091	17.45	191
3	4.2	1.25 × 10^−5^	9.62 × 10^−3^	769	2.46 × 10^−5^	0.028	1138	0.138	25.58	185
	3.84	1.15 × 10^−5^	1.32 × 10^−2^	1148	1.88 × 10^−5^	0.025	1329	0.105	21.83	208
	3.8	1.13 × 10^−5^	9.28 × 10^−3^	821	1.82 × 10^−5^	0.021	1154	0.102	21.88	214
	3.44	1.03 × 10^−5^	1.27 × 10^−2^	1233	1.35 × 10^−5^	0.018	1333	0.076	18.42	242
3.5	4.2	1.07 × 10^−5^	0.014	1308	1.55 × 10^−5^	0.021	1355	0.118	26.73	226
	3.84	9.82 × 10^−6^	0.019	1935	1.18 × 10^−5^	0.018	1525	0.09	23.04	256
	3.8	9.72 × 10^−6^	0.013	1337	1.15 × 10^−5^	0.016	1391	0.087	23.04	265
	3.44	8.79 × 10^−6^	0.018	2048	8.5 × 10^−6^	0.012	1412	0.065	19.56	301

## Discussion

We have developed a serpentine shaped polyimide electrode cable that is nearly one order of magnitude more compliant than a straight polyimide cable of same dimensions. The design parameters were carefully evaluated using analytical and numerical models. Prototypes of the cables were micro-fabricated using MEMS microfabrication techniques. Fabricated prototypes were subjected to in-plane and out-of-plane stress experiments and the compliance was measured. Measured compliance of the serpentine cable was 6–10 times higher than the compliance of the straight cable.

It is expected that a 10-fold increase in electrode interconnect compliance may enable more reliable chronic neural recording even from awake animals and the electrophysiological recordings are expected to have better signal-to-noise ratio and impedance. However, *in vivo* experiments are needed to validate and quantify this. Though increased compliance in all three axes will be useful for reduced stain, it is important to have more compliance in the *y*-axis since the relative displacement of the brain with respect to the skull will be highest in that direction (16). The new serpentine cables are shown to be 10 times more compliant in the *y*-axis, which will be critical in *in vivo* studies.

It should be noted that the Young’s modulus of polyimide (3–15 GPa) is much less than that of silicon (200 GPa). With one order of magnitude less Young’s modulus, the compliance of the polyimide cables is expected to be much higher than silicon ribbon cables. Furthermore, the serpentine structure provides additional flexibility to the interconnect. However, polyimide has a water absorption rate of ∼1.1%. *In vitro* study of polyimide as a long-term implantable material has shown that polyimide of different formulations undergo changes in crystalline structure and mechanical properties due to constant water uptake (34). The changes in mechanical properties include increase in Young’s modulus and decrease in tensile strength. The results from the study predict that the constant uptake of water for over 20 months could act as a plasticizer and can increase the stiffness of the material. Based on these observations, it can be implied that the compliance of the cables will decrease in chronic conditions due to constant moisture uptake. Similarly, the cables will undergo additional stress due to biological and chemical reactions, and other complex mechanical motions in *in vivo* conditions, which may potentially affect the compliance. It is expected that the serpentine geometry will compensate for any increase in the cable stiffness. Further studies are needed to evaluate the chronic *in vivo* behavior of these polyimide serpentine cables.

It can be noted from Tables 3 and 4 that there is some discrepancy between the analytical and the numerical results and the experimentally measured values. This discrepancy could have resulted from the assumptions made for the analytical and numerical analyses and from the limitations in the geometry of the finite element solver. A clamped-guided beam model was assumed for the analytical and the numerical analyses. The boundary conditions of this model allow the guided end to deflect normal to its axis, while restricting its rotational motion. However in practice, there will be some rotational motion displayed by the beam which will contribute to the overall beam deflection. Furthermore, in the case of the serpentine structure, the meanders will have flexural degrees of freedom which will be different from the rigid body degrees of freedom, and there will be an additional effect of beam twisting seen in the serpentine cables.

In addition to its compliance, the proposed cable design also permits the placement of the recording microwires in a transverse fashion thereby providing a 2D recording space for the array. The 2D high compliance serpentine electrode arrays are expected to provide strain relief for the recording microwires and in turn, mitigate the strain induced tissue response. With a reduction in the tissue response, the implant can be expected to have improved performance in chronic applications.

## Conflict of Interest Statement

The authors declare that the research was conducted in the absence of any commercial or financial relationships that could be construed as a potential conflict of interest.

## Supplementary Material

The Supplementary Material for this article can be found online at http://www.frontiersin.org/Neuroprosthetics/10.3389/fneur.2013.00124/abstract

## Footnotes

^1^http://abaqus.me.chalmers.se/v6.12/books/usb/default.htm?startat=pt03ch07s02aus51.html

^2^5960 Series: Dual Column Tabletop (5–50 kN) manual, Instron Inc

^3^TI 900 TriboIndenter manual, Hysitron Inc

## References

[1] Polikov V Tresco P Reichert W Response of brain tissue to chronically implanted neural electrodes. J Neurosci Methods(2005) 148(1):1–18 10.1016/j.jneumeth.2005.08.015 16198003

[2] Prasad A Sankar V Dyer AT Knott E Xue Q-S Nishida T Coupling biotic and abiotic metrics to create a testbed for predicting neural electrode performance. Conf Proc IEEE Eng Med Biol Soc (2011) 2011:3020–3 22254976 10.1109/IEMBS.2011.6090827

[3] Streit WJ Xue Q-S Prasad A Sankar V Knott E Dyer A Tissue, electrical, and material responses in electrode failure. IEEE Pulse (2012) 3(1):30–3 10.1109/MPUL.2011.2175632 22344948

[4] Prasad A Xue Q-S Sankar V Nishida T Shaw G Streit WJ Comprehensive characterization and failure modes of tungsten microwire arrays in chronic neural implants. J Neural Eng (2012) 9(5):056015 10.1088/1741-2560/9/5/056015 23010756

[5] Hochberg L Serruya M Friehs G Mukand J Saleh M Caplan A Neuronal ensemble control of prosthetic devices by a human with tetraplegia. Nature (2006) 442(7099):164–71 10.1038/nature04970 16838014

[6] Simeral J Kim S Black M Donoghue J Hochberg L Neural control of cursor trajectory and click by a human with tetraplegia 1000 days after implant of an intracortical microelectrode array. J Neural Eng (2011) 8(2):025027 10.1088/1741-2560/8/2/025027 21436513 PMC3715131

[7] Hochberg L Bacher D Jarosiewicz B Masse N Simeral J Vogel J Reach and grasp by people with tetraplegia using a neurally controlled robotic arm. Nature (2012) 485(7398):372–5 10.1038/nature11076 22596161 PMC3640850

[8] Lebedev M Nicolelis M Brain-machine interfaces: past, present and future. Trends Neurosci (2006) 29(9):536–46 10.1016/j.tins.2006.07.004 16859758

[9] Nicolelis MAL Lebedev MA Principles of neural ensemble physiology underlying the operation of brain-machine interfaces. Nat Rev Neurosci (2009) 10:530–40 10.1038/nrn2653 19543222

[10] Lebedev MA Tate AJ Hanson TL Li Z O’Doherty JE Winans JA Future developments in brain-machine interface research. Clinics (2011) 66(S1):25–32 10.1590/S1807-59322011001300004 21779720 PMC3118434

[11] Biran R Martin D Tresco P The brain tissue response to implanted silicon microelectrode arrays is increased when the device is tethered to the skull. J Biomed Mater Res A (2007) 82(1):169–78 17266019 10.1002/jbm.a.31138

[12] Kim Y Hitchcock R Bridge M Tresco P Chronic response of adult rat brain tissue to implants anchored to the skull. Biomaterials (2004) 25(12):2229–37 10.1016/j.biomaterials.2003.09.010 14741588

[13] Biran R Martin D Tresco P Neuronal cell loss accompanies the brain tissue response to chronically implanted silicon microelectrode arrays. Exp Neurol (2005) 195(1):115–26 10.1016/j.expneurol.2005.04.020 16045910

[14] Chestek CA Gilja V Nuyujukian P Foster JD Fan JM Kaufman MT Long-term stability of neural prosthetic control signals from silicon cortical arrays in rhesus macaque motor cortex. J Neural Eng (2011) 8:045005 10.1088/1741-2560/8/4/045005 21775782 PMC3644617

[15] Gilletti A Muthuswamy J Brain micromotion around implants in the rodent somatosensory cortex. J Neural Eng (2006) 3(3):189–95 10.1088/1741-2560/3/3/001 16921202

[16] Lee H Bellamkonda R Sun W Levenston M Biomechanical analysis of silicon microelectrode-induced strain in the brain. J Neural Eng (2005) 2(4):81–9 10.1088/1741-2560/2/4/003 16317231

[17] Subbaroyan J Martin D Kipke D A finite-element model of the mechanical effects of implantable microelectrodes in the cerebral cortex. J Biomed Mater Res A (2005) 2(4):103–13 16317234 10.1088/1741-2560/2/4/006

[18] Subbaroyan J Kipke D The role of flexible polymer interconnects in chronic tissue response induced by intracortical microelectrodes – a modeling and an in vivo study. Conf Proc IEEE Eng Med Biol Soc (2006) 1:3588–91 17947041 10.1109/IEMBS.2006.260517

[19] Rousche P Pellinen D Pivin DP Jr. Williams J Vetter R Kipke D Flexible polyimide-based intracortical electrode arrays with bioactive capability. IEEE Trans Biomed Eng (2001) 48(3):361–71 10.1109/10.914800 11327505

[20] Stieglitz T Gross M Flexible BIOMEMS with electrode arrangements on front and back side as key component in neural prostheses and biohybrid systems. Sens Actuators B Chem (2002) 83(1–3):8–14 10.1016/S0925-4005(01)01021-8

[21] Stieglitz T Schuetter M Koch K Implantable biomedical microsystems for neural prostheses. IEEE Eng Med Biol Mag (2005) 24(5):58–65 10.1109/MEMB.2005.1511501 16248118

[22] Takeuchi S Suzuki T Mabuchi K Fujita H 3D flexible multichannel neural probe array. J Micromech Microeng (2004) 14(1):104 10.1088/0960-1317/14/1/014

[23] Pellinen D Moon T Vetter R Miriani R Kipke D Multifunctional flexible parylene-based intracortical microelectrodes. Conf Proc IEEE Eng Med Biol Soc (2005) 5:5272–5 17281439 10.1109/IEMBS.2005.1615669

[24] Seymour J Kipke D Neural probe design for reduced tissue encapsulation in CNS. Biomaterials (2007) 28:3594–607 10.1016/j.biomaterials.2007.03.024 17517431

[25] Salcman M Bak M Design, fabrication, and in vivo behavior of chronic recording intracortical microelectrodes. IEEE Trans Biomed Eng (1973) 20(4):253–60 10.1109/TBME.1973.324189 4708761

[26] Yao Y Gulari M Casey B Wiler J Wise K Silicon microelectrodes with flexible integrated cables for neural implant applications. In: International IEEE/EMBS Conference on Neural Engineering Kohala Coast: IEEE (2007). p. 398–401 10.1109/CNE.2007.369693

[27] Gilgunn PJ Khilwani R Kozai TDY Weber DJ Cui T Erdos G An ultra-compliant scalable neural probe with molded biodissolvable delivery vehicle. IEEE 25th International Conference MEMS Paris: IEEE (2012). p. 56–9 10.1109/MEMSYS.2012.6170092

[28] Kim E Tu H Lv C Jiang H Yu H Xu Y A robust polymer microcable structure for flexible devices. Appl Phys Lett (2013) 102:033506 10.1063/1.4788917

[29] Patrick E Sankar V Rowe W Yen S-F Sanchez JC Nishida T Flexible polymer substrate and tungsten microelectrode array for an implantable neural recording system. Conf Proc IEEE Eng Med Biol Soc (2008) 2008:3158–61 10.1109/IEMBS.2008.4649874 19163377

[30] Patrick E Sankar V Rowe W Sanchez JC Nishida T An implantable integrated low-power amplifier-microelectrode array for brain-machine interfaces. Conf Proc IEEE Eng Med Biol Soc (2010) 2010:1816–9 10.1109/IEMBS.2010.5626419 21095940

[31] Barillaro G Molfese A Nannini A Pieri F Analysis, simulation and relative performances of two kinds of serpentine springs. J Micromech Microeng (2005) 15(4):736–46 10.1088/0960-1317/15/4/010

[32] Fedder GK Simulations of Microelectromechanical Systems [Dissertation]. Berkeley: University of California at Berkeley (1994).

[33] Dolbow J Gosz M Effect of out-of-plane properties of a polyimide film on the stress fields in microelectronic structures. Mech Mater (1996) 23:311–21 10.1016/0167-6636(96)00021-X

[34] Rubehn B Stieglitz T In vitro evaluation of the longterm stability of polyimide as a material for neural implants. Biomaterials (2010) 31(13):3449–58 10.1016/j.biomaterials.2010.01.053 20144477

