# Supplementary material for: A Highly Compliant Serpentine Shaped Polyimide Interconnect for Front-End Strain Relief in Chronic Neural Implants
Source: Front Neurol. 2013 Sep 12;4:124. doi: 10.3389/fneur.2013.00124 (PMC3770980; doi:10.3389/fneur.2013.00124)
Supplement: Supplementary file 1 [file 55721_Nishida_DataSheet1.PDF]

Analytical Analysis of Cable Compliance for Different Lengths and Widths

Comparison of the compliances of short straight and serpentine cables (cable length L < 6 mm)

(i) Cable width = 2.5 mm

Overall cable length	X-axis			Y-axis			Z- axis		
	Straight cable compliance (m/N)	Serpentine cable Compliance (m/N)	Increase in compliance of serpentine cable	Straight cable compliance (m/N)	Serpentine cable Compliance (m/N)	Increase in compliance of serpentine cable	Straight cable compliance (m/N)	Serpentine cable Compliance (m/N)	Increase in compliance of serpentine cable
L = 4.2 mm	1.50×10^{-5}	6.29×10^{-3}	**419 times**	4.24×10^{-5}	0.034	**801 times**	0.166	24.57	**148 times**
L = 3.84 mm	1.37×10^{-5}	8.59×10^{-3}	**627 times**	3.24×10^{-5}	0.032	**987 times**	0.127	20.79	**163 times**
L = 3.8 mm	1.36×10^{-5}	6.04×10^{-3}	**444 times**	3.14×10^{-5}	0.027	**860 times**	0.122	20.92	**171 times**
L = 3.44 mm	1.23×10^{-5}	8.24×10^{-3}	**670 times**	2.33×10^{-5}	0.025	**1072 times**	0.091	17.45	**191 times**

Comparison of the compliances of short straight and serpentine cables (cable length L < 6 mm)

(ii) Cable width = 3 mm

Overall cable length	X-axis			Y-axis			Z- axis		
	Straight cable compliance (m/N)	Serpentine cable Compliance (m/N)	Increase in compliance of serpentine cable	Straight cable compliance (m/N)	Serpentine cable Compliance (m/N)	Increase in compliance of serpentine cable	Straight cable compliance (m/N)	Serpentine cable Compliance (m/N)	Increase in compliance of serpentine cable
L = 4.2 mm	1.25×10^{-5}	9.62×10^{-3}	**769 times**	2.46×10^{-5}	0.028	**1138 times**	0.138	25.58	**185 times**
L = 3.84 mm	1.15×10^{-5}	0.0132	**1148 times**	1.88×10^{-5}	0.025	**1329 times**	0.105	21.83	**208 times**
L = 3.8 mm	1.13×10^{-5}	9.28×10^{-3}	**821 times**	1.82×10^{-5}	0.021	**1154 times**	0.102	21.88	**214 times**
L = 3.44 mm	1.03×10^{-5}	0.0127	**1233 times**	1.35×10^{-5}	0.018	**1333 times**	0.076	18.42	**242 times**

Comparison of the compliances of short straight and serpentine cables (cable length L < 6 mm)

(iii) Cable width = 3.5 mm

Overall cable length	X-axis			Y-axis			Z- axis		
	Straight cable compliance (m/N)	Serpentine cable Compliance (m/N)	Increase in compliance of serpentine cable	Straight cable compliance (m/N)	Serpentine cable Compliance (m/N)	Increase in compliance of serpentine cable	Straight cable compliance (m/N)	Serpentine cable Compliance (m/N)	Increase in compliance of serpentine cable
L = 4.2 mm	1.07 x 10-5	0.014	**1308 times**	1.55 x 10-5	0.021	**1355 times**	0.118	26.73	**226 times**
L = 3.84 mm	9.82 x 10-6	0.019	**1935 times**	1.18 x 10-5	0.018	**1525 times**	0.09	23.04	**256 times**
L = 3.8 mm	9.72 x 10-6	0.013	**1337 times**	1.15 x 10-5	0.016	**1391 times**	0.087	23.04	**265 times**
L = 3.44 mm	8.79 x 10-6	0.018	**2048 times**	8.5 x 10-6	0.012	**1412 times**	0.065	19.56	**301 times**
